# Hepatitis B Virus Integration into Transcriptionally Active Loci and HBV-Associated Hepatocellular Carcinoma

**DOI:** 10.3390/microorganisms10020253

**Published:** 2022-01-24

**Authors:** Maria Bousali, Timokratis Karamitros

**Affiliations:** 1 Bioinformatics and Applied Genomics Unit, Department of Microbiology, Hellenic Pasteur Institute, 11521 Athens, Greece; mbousali@med.uoa.gr; 2 Laboratory of Medical Microbiology, Department of Microbiology, Hellenic Pasteur Institute, 11521 Athens, Greece

**Keywords:** hepatitis B virus, viral integration, VIS, pathogen–host interactions, hepatocellular carcinoma, HBV-HCC

## Abstract

Hepatitis B Virus (HBV) DNA integrations into the human genome are considered major causative factors to HBV-associated hepatocellular carcinoma development. In the present study, we investigated whether HBV preferentially integrates parts of its genome in specific genes and evaluated the contribution of the integrations in HCC development per gene. We applied dedicated in-house developed pipelines on all of the available HBV DNA integration data and performed a statistical analysis to identify genes that could be characterized as hotspots of integrations, along with the evaluation of their association with HBV-HCC. Our results suggest that 15 genes are recurrently affected by HBV integrations and they are significantly associated with HBV-HCC. Further studies that focus on HBV integrations disrupting these genes are mandatory in order to understand the role of HBV integrations in clonal advantage gain and oncogenesis promotion, as well as to determine whether inhibition of the HBV-disrupted genes can provide a therapy strategy for HBV-HCC.

## 1. Introduction

The hepatitis B virus (HBV) is a 3200 bp length hepatotropic virus of the Hepadnaviridae family and is classified into Group VII in Baltimore’s system as it combines a partially double-stranded DNA (ds-DNA) and virus-encoded reverse transcriptase. The “Out of Africa” model for the origin and expansion of HBV has been supported in previous studies [1,2] and the origin of HBV in *Homo sapiens* has been estimated at 34,100 (27,600–41,300) years ago [3].

It has been found, since the early 1980s, that HBV integrates its genome, or parts of it, into the human genomic DNA [4,5,6,7], while more recent findings suggest that HBV integrations occur in the infected hepatocytes during the early stages of natural acute infections [8,9,10,11]. Multiple molecular mechanisms have been proposed to be involved in the integration process [12], of which the most prevalent are the non-homologous end joining (NHEJ) and the microhomology-mediated end joining (MMEJ) [13,14].

HBV infection causes chronic hepatitis and, subsequently, liver cirrhosis, while it has been identified as one of the most important contributors of hepatocellular carcinoma (HCC), as ~50% of the cases are HBV-associated (HBV-HCC) [15]. Moreover, the integrated HBV DNA is a continual source of HBV subviral particles, and the HBsAg they contain chronically inhibits the innate and adaptive immune responses to HBV infection [14,16,17,18,19,20]. This is due to the change of the organization of the HBV genome from circular to linear after the integration, preventing the expression of the HBV core, the polymerase, the E-antigen, and, frequently, the large isoform form of HBsAg and X-antigen, as the pregenomic RNA is not produced [14,20].

Retroviral and infectious viral integration events induce chromosome changes, genome instability, changes in the expression of host genes, and oncogenes activation, as well as inflammation [21]. Thus, HBV integrations are critical factors in liver disease progression and HCC development, having been detected in more than 85% of HBV-HCC cases [4,22,23,24].

Previous independent studies suggest that genomic areas that refer to cellular genes are favored target sites for HBV integration [10], and, specifically, that there is a preference for HBV integration into transcriptional and chromosomal regulatory regions [25]. Moreover, it has been reported that known HCC driver genes, such as the *telomerase reverse transcriptase* (*TERT*), the *lysine methyltransferase 2B* (*KMT2B*—also known as *MLL4*), and the *cyclin E1* (*CCNE1*), are affected by HBV integrations in HBV-HCC cases [26].

In this study, we investigated whether HBV preferentially integrates its genome or parts of it into specific transcriptionally active genomic loci, and evaluated the contribution of each integration in HCC development. We applied dedicated in-house developed pipelines on all of the available HBV DNA integration sites data and performed statistical analyses to identify genes that could be characterized as hotspots of HBV integrations.

## 2. Methods

**Figure 1:**
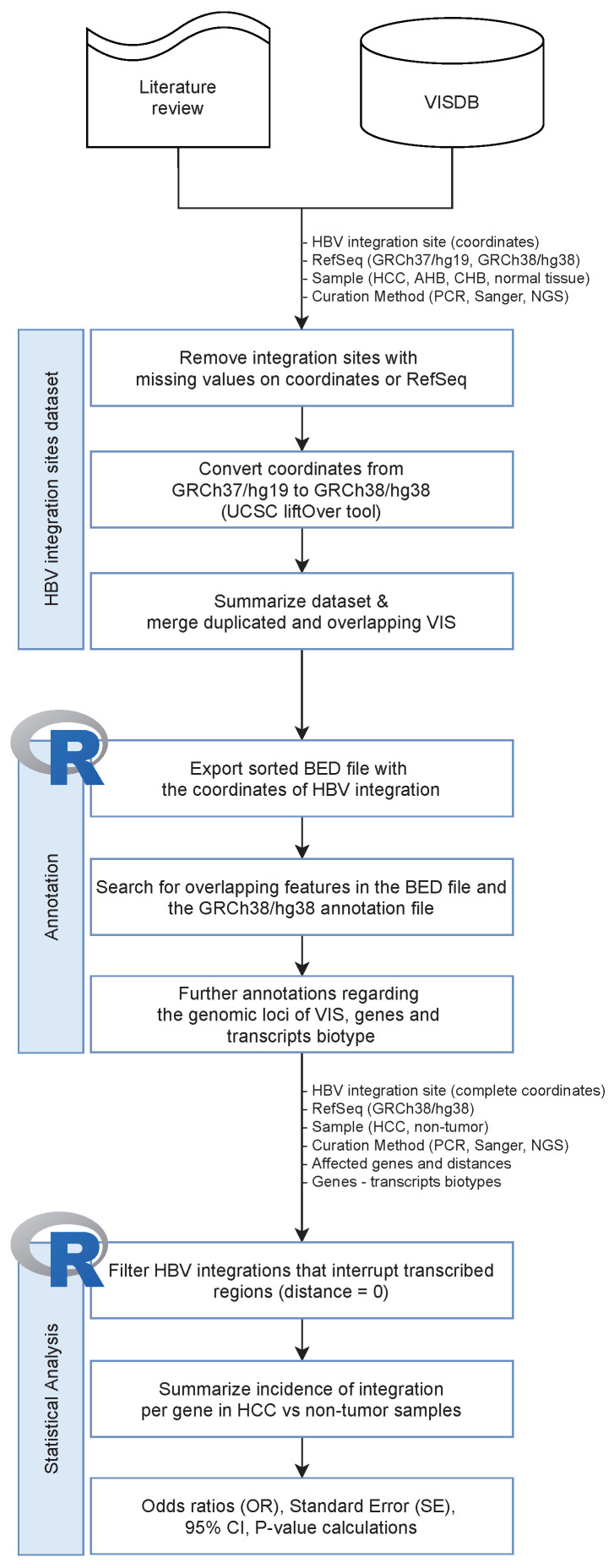
Multi-step workflow diagram of the methodology used to study the effect of HBV DNA integrations in transcribed genomic loci of the human genome (RefSeq GRCh38/hg38). Data regarding HBV DNA integration sites (coordinates) into human samples, the reference sequences used in the initial analysis, and the type of samples (HCC, AHB, CHB, normal tissue) used to export the viral integration sites (VIS), as well as wet-lab methodology information, were mined from 30 research papers and merged with additional metadata that are present in the VISDB database [54]. The dataset was curated: VIS coordinates for which both the start and end positions were absent, or the human reference build was not reported, were removed from the dataset, coordinates that referred to the GRCh37/hg19 human genome reference were converted to GRCh38/hg38 with the UCSC LiftOver tool [55], and duplicated and overlapping VIS were merged. The annotation of the VIS-disrupted genomic loci was performed with in-house developed scripts written in R language by applying a three-step approach that involves the generation of a sorted BED-formatted file with the curated VIS, the identification of overlapping regions between VIS and the GRCh38/hg38 annotation file, and the identification of the corresponding genes and transcripts biotypes. The annotated dataset was filtered in order to include only VIS that disrupt transcriptionally active regions, and the incidence of integrations in HCC and non-HCC samples was summarized per gene. The statistical analysis was based on per-gene calculations of the odds ratios (OR) between HCC and non-HCC samples; the standard error of OR, the 95% CI, and statistical significance was declared at a *p*-value < 0.01.

The coordinates of the human genome that have been reported to correspond to HBV DNA integration sites in human samples (excluding integrations reported to occur in cell lines), as well as metadata regarding the dataset characteristics, the originating samples (HCC, acute HBV infection—AHB, chronic HBV infection—CHB, or non-tumor adjacent tissue samples), and the curation method of the integration sites, were retrieved from the from 30 research articles [10,13,24,27,28,29,30,31,32,33,34,35,36,37,38,39,40,41,42,43,44,45,46,47,48,49,50,51,52,53] and the VISDB database [54]. In total, 20,153 HBV integration sites were evaluated (Figure 1). HBV integration sites that are registered to VISDB and have missing values in the start and the end positions of their genomic coordinates, or for which the human reference build is not reported, were removed, resulting in 19,600 integration sites.

The coordinates referring to the GRCh37/hg19 human genome reference (19,068/19,600), were converted to GRCh38/hg38 using the UCSC LiftOver tool [55]. From them, one coordinate was marked as a “lift over failure” as it has been deleted in GRCh38/hg38 reference build. After the conversion, 3267 coordinates were found to be duplicated or overlapping and were removed, resulting in 16,332 HBV integration sites, from which 16,313 are located in chromosomes 1-22, X, Y, and 19 in alternative loci scaffolds. These integrations were obtained from 4564 human samples, from which 2448 were HCC samples obtained from HCC tissues resected from patients undergoing primary hepatectomy and 2116 were non-tumor samples. The non-tumor samples category includes samples obtained either from matched adjacent tissues of the HCC samples or from liver biopsy specimens collected from patients with AHB and CHB.

The genomic loci referring to HBV integrations were annotated by in-house developed scripts written in R programming language [56], using the tidyverse [57] and the biomaRt [58,59] libraries.

Assuming that HBV integration is a random event and that the genomic locus of the integration depends on the size of each chromosome, the expected rate of HBV integration into each chromosome was calculated, as described by Minami et al. [60]. A binomial test was employed to analyze the frequency of integration per chromosome and the statistical significance was declared at a *p*-value < 0.01. Afterward, the annotated dataset was filtered in order to focus on integrations that interrupt transcriptionally active genomic regions (distance from gene = 0), and the incidence of integrations in HCC and non-tumor samples was summarized per gene. Gene ontology (GO) and other functional enrichment analyses (KEGG, REAC, CORUM) were performed with R programming language using the gprofiler2 library [61].

Odds ratios (OR) together with 95% confidence intervals (CI) were used to estimate the strength of association between the HBV DNA integrations and HCC, as previously described in [62,63,64]. In brief, the OR was calculated as the ratio between the odds of HBV integrations occurring in HCC samples and the odds of HBV integrations occurring in non-tumor samples. The standard error (SE) of the OR was calculated as the square root of the sum of the inverse of the four different observed states (HCC samples with integrations, non-tumor samples with integrations, HCC samples without integrations, and non-tumor samples without integrations). The 95% confidence intervals (CI) were calculated through the equation $e^{\left[log(OR)\pm 1.96\times SE\right]}$, where OR is the odds ratio and SE is the standard error of the OR. For the calculation of the *p*-value from the 95% CI, the procedure described by Altman et al. [65] was followed. The statistical significance was declared at a *p*-value < 0.01.

## 3. Results and Discussion

**Figure 2:**
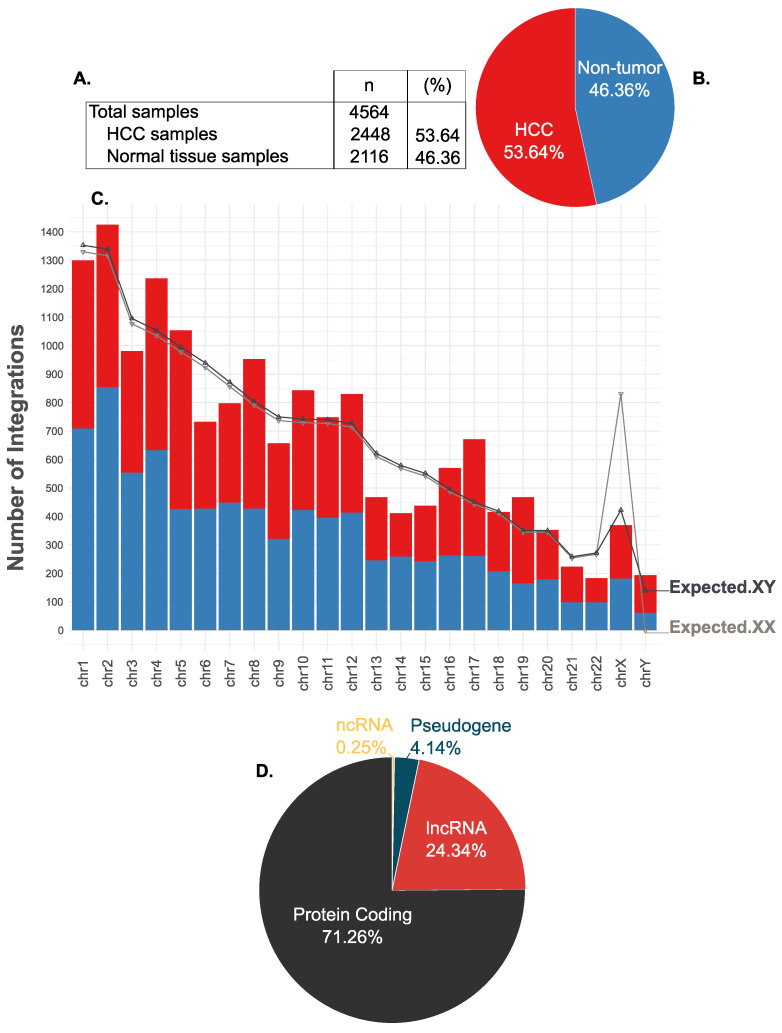
Graphical representation of the characteristics of the dataset used in the present study, as well as the findings obtained from the annotation of the HBV integration sites. (**A**,**B**) The total samples from which the HBV integrations are analyzed in the present study were obtained from previously published research papers and correspond to 2448 (53.64% of the total samples) HCC samples and 2116 (46.36% of the total samples) non-tumor tissue samples (total number of samples = 4564). (**C**) The distribution of HBV integrations in the human chromosomes (chr1–22, chrX, chrY) are depicted as bar charts. Moreover, the distribution of the HBV integrations in HCC and non-tumor samples are also depicted in the stacked bar charts, in red and blue, respectively. The expected values used in the binomial test, in which it was assumed that HBV integration is a random event and that the genomic locus of the integration depends on the size of each chromosome, are presented as lines with gray color and the (▽) symbol for the 46XX subjects and black color and the (△) symbol for the 46XY subjects. (**D**) Representation of the biotypes of the genes that are being disrupted from HBV integration. From the 5053 disrupted, transcribed genomic loci, 3601 (71.26%) are protein-coding genes, 1230 (24.34%) are long non-coding RNAs (lncRNA), 209 (4.14%) are pseudogenes, and 13 (0.25%) are non-coding RNAs (ncRNA).

The HBV DNA integrations evaluated in the present study were obtained from 4564 samples that have been analyzed in independent research studies, from which 2448 (53.46%) are HCC samples and 2116 (46.54%) are non-tumor tissue samples (Figure 2A,B).

Binomial tests were performed in order to evaluate if there is a preference or a deterrence for HBV integration to specific chromosomes (Table 1, Figure 2C). It was demonstrated that integrations in chromosomes 4, 8, 10, 12, 16, 17, and 19 were higher than anticipated and the overall frequencies were found to be statistically significant (*p* < 0.01). On the other hand, integrations in chromosomes 3, 6, 9, 13, 14, 15, and 22 were lower than anticipated and the overall frequencies were found to be statistically significant (*p* < 0.01). Previous independent studies have reported the preference for HBV integrations to chromosomes 3 [60], 11 [66], and 17 [33]. In this study, it is revealed that there is indeed a preference for integrations in chromosome 17 and a deterrence for integration for chromosome 3. These observations suggest that HBV integration is not a random event, as the genomic locus of the integration does not depend only on the size of each chromosome. As it has been previously suggested [60], selective pressure may be applied. Other factors, such as microhomology regions between the HBV and the human genome, along with the molecular mechanisms that initiate the integration process, should be further studied in order to gain more insights and explain the observed preference/deterrence of integrations into these chromosomes. Recent studies have reported that HBV frequently integrates into mobile genetic elements, such as transposons and retrotransposons, with a preference for the latter [11,67,68]. These findings form evidence for a (retro)transposon-directed integration process, which may have a potential role in the observed nonrandom HBV integration.

Figure 2D represents the biotypes of the genes that are disrupted from HBV integrations. From the 5053 transcribed genomic loci that are being disrupted from HBV integrations, 3601 (71.26%) are protein-coding genes, 1230 (24.34%) are long non-coding RNAs (lncRNA), 209 (4.14%) are pseudogenes, and 13 (0.25%) are non-coding RNAs (ncRNA).

**Figure 3:**
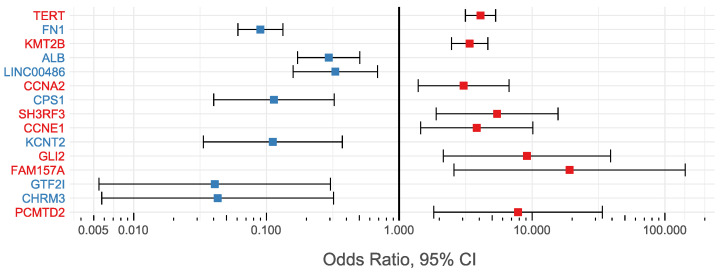
Odds ratios (OR) and 95% confidence intervals (95% CI) obtained by comparing the HBV integrations between the HCC and non-tumor groups in 15 human genes, represented as a log${}_{10}$-scaled plot.

Table 2 and Figure 3 present the results of the comparison of HBV DNA integration in HCC between the HCC and non-tumor groups. The overall results suggest that integrations into the *TERT*, the *KMT2B*, the *CCNA2*, the *SH3RF3*, the *CCNE1*, the *GLI2*, the *FAM157A*, and the *PCMTD2* genes are more common in HCC samples than in non-tumor samples (OR > 1, *p*-value < 0.01), while the opposite is true for the *FN1*, the *ALB*, the *LINC00486*, the *CPS1*, the *KCNT2*, the *GTF2I*, and the *CHRM3* genes (OR < 1, *p*-value < 0.01). Based on this evidence, integrations disrupting the *TERT* and the *KMT2B* genes are more likely to impact the HCC development. These two genes have been found as major genomic locations of recurrent HBV DNA integrations (present in 12.75% and 7.72% of the total HCC samples, respectively) and possibly have a positive association with HCC (OR > 1, *p*-value < 0.01).

The *TERT* gene contributes to the formation of the telomerase enzyme whose role is the maintenance of the telomeres—the “protectors” of the chromosomes from abnormal sticking together and degradation. In the hepatocytes, telomeres are progressively shortened because of cell division, and when they reach a critical length, they trigger the cell to stop division and undergo apoptosis. Moreover, the majority of the cancer cells (80–90%) activate the *TERT* gene in order to achieve immortality [69,70,71]. HBV integrations into the *TERT* gene are found in 12.75% of the HCC samples in the dataset. Thus, they should be further studied in order to determine the possible role of integrations in the gain of clonal advantage that results in HBV-HCC. Further studies will also help in clarifying whether the inhibition of the HBV-disrupted *TERT* can provide a therapy strategy for HBV-HCC, as it has been suggested for a wide spectrum of cancers [72,73]. Along with *TERT*, the *CCNA2* and the *CCNE1* genes are involved in the cell cycle G1/S transition (GO:0044843). Misregulation of this biological process has been associated with oncogenesis promotion [74]. The two cyclins, *CCNA2* and *CCNE1*, have been found as major genomic locations of recurrent HBV DNA integrations (present in 0.79% and 0.59% of the total samples, respectively), and integrations that disrupt them possibly have a positive association with HCC (OR = 3.049 and 3.829, respectively, *p*-value < 0.01). These results are in concordance with previous studies that mention that the *TERT* and *CCNE1* have oncogenetic or tumor-suppressing functions [13].

*KMT2B* has been described as an oncogene, as well as a tumor-suppressor gene, that helps in the prevention of uncontrolled cell growth and division [75]. HBV DNA integrations into it are present in 7.72% of the HCC samples in the dataset and there is a possible positive association with HCC (OR = 3.388, *p*-value < 0.01).

On the other hand, *FN1* and *ALB* genes that are recurrently affected by HBV integrations (6.09% and 5.26% of the total samples in the dataset) have been found to be disrupted in 1.18% and 0.74% of HCC samples versus 11.77% and 2.46% of the non-tumor samples and they are possibly negatively associated with HCC (OR = 0.09 and 0.294, respectively, *p*-value < 0.01). The observation that FN1 is recurrently affected by HBV integration in non-tumor samples is in concordance with previous findings [31]. A reasonable explanation could be based on the fact that *FN1* has been described as an oncogene, but this has not been evaluated. HBV integrations that disrupt the *FN1* and *ALB* genes possibly do not endow the carrier hepatocytes with a selective growth advantage, but this hypothesis should, also, be further investigated. Although, due to the fact that no follow-up was performed in the cases from which non-tumor liver sample was taken for the analysis, it cannot be ruled out that these integrations may be a prognostic factor for HCC, as discussed below.

At the same time, HBV integrations in four recurrently disrupted genes [12], *LINGO2*, *PRKN*, *SOX5*, and *PDE3A*, were not found to be statistically significant (*p*-value > 0.01). In brief, the *LINGO2* gene was disrupted from HBV integrations in 25 samples (0.55% of the total samples in the dataset), from which 13 were HCC samples (0.53% of the total HCC samples) and 12 were non-tumor samples (0.57% of the total non-tumor samples). The *PRKN* gene was disrupted in 23 samples (0.55%), from which 14 were HCC samples (0.57%) and 9 were non-tumor samples (0.43%). The *SOX5* gene was disrupted in 22 samples (0.48%), from which 7 were HCC samples (0.29%) and 15 were non-tumor samples (0.71%), while the *PDE3A* gene was disrupted in 22 samples (0.48%), from which 14 were HCC samples (0.57%) and 8 were non-tumor samples (0.38%).

To conclude, the present study provides new insights into the impact of HBV integrations that disrupt transcribed genomic loci on HCC development, by combining all of the available knowledge obtained from independent published research studies. The findings provided suggest that 15 genes are recurrently affected by HBV integrations and they are significantly associated with HBV-HCC, and thus may represent HCC driver genes that merit further investigation as to their possible role in therapeutical targets for HBV-HCC and molecular profiling for precision medicine. At the same time, HBV integration was found to be a non-random event, thus selective pressure may be applied, while the role of microhomologies between HBV and the human genome, as well as the molecular mechanisms initiating the integration process—including the arising evidence for (retro)transposon-mediated integration—are matters demanding further investigation. The present study included manually curated integration sites, as are those provided by the VISDB database [54], thus the biases occurring from using data from independent research studies, whose results have been exported from different methodologies, were minimized. A systematic review regarding the HBV DNA integrations has previously been conducted [12] and all available data on a topic have been summarized (reviewer selection bias minimization). It should be noted though, that cases with CHB or AHB from which samples were taken and analyzed were not followed up. Thus, integrations that are found more frequently in non-tumor samples and are not associated with tumorigenesis warrant further studies investigating their role and whether they can be used as a prognostic risk factor of tumor development. Moreover, other factors, such as the alanine aminotransferase (ALT) levels, the serum HBV DNA levels, the circulating cell-free DNA (cfDNA) [76], the level of liver fibrosis, the heterogeneity of the tumor samples, and other genomic polymorphisms [77] that could be possibly found in the samples, whether cases underwent antiviral therapy before the resection surgery [78], or the presence of anti-HBe, anti-HBs, or anti-HBc, may be confusing factors of the observed associations and should be co-evaluated in future prospective studies on HBV VIS. Further studies are mandatory to better understand the association of HBV integrations with HBV-HCC, the molecular mechanisms that mediate in the integration process, and to define the possibility to prevent or treat HCC induced by HBV DNA integrations.

## Figures and Tables

**Table 1:** Chromosomal location of HBV integrations and evaluation of possible preference or deterrence to specific chromosomes by binomial tests (*p* < 0.01), assuming that HBV integration is a random event and that the genomic loci of the integration depend on the size of each chromosome.

chr	Size (Mb)	Frequency	Expected.XX	Expected.XY	*p*-Value	Explanation
chr1	246.1	1299	1329.52	1352.68	0.146	insignificant
chr2	243.6	1425	1316.02	1338.94	0.019	insignificant
chr3	199.3	981	1076.69	1095.45	0	deterrence
chr4	191.7	1236	1035.63	1053.68	0	preference
chr5	181.0	1054	977.83	994.86	0.061	insignificant
chr6	170.9	732	923.27	939.35	0	deterrence
chr7	158.5	797	856.28	871.19	0.012	insignificant
chr8	146.3	953	790.37	804.13	0	preference
chr9	136.4	657	736.88	749.72	0.001	deterrence
chr10	135.0	843	729.32	742.02	0	preference
chr11	134.5	748	726.62	739.28	0.741	insignificant
chr12	132.1	830	713.65	726.08	0	preference
chr13	113.0	467	610.47	621.10	0	deterrence
chr14	105.3	411	568.87	578.78	0	deterrence
chr15	100.3	437	541.86	551.30	0	deterrence
chr16	90.0	570	486.21	494.68	0.001	preference
chr17	81.9	671	442.45	450.16	0	preference
chr18	76.1	415	411.12	418.28	0.903	insignificant
chr19	63.8	467	344.67	350.68	0	preference
chr20	63.7	352	344.13	350.13	0.915	insignificant
chr21	47.0	223	253.91	258.33	0.027	insignificant
chr22	49.4	183	266.88	271.53	0	deterrence
chrX	153.7	369	830.34	422.40	0.009	deterrence
chrY	50.3	193	0.00	138.24	0	preference

**Table 2:** Association between HBV DNA integrations and HCC in 15 target human genes.

Gene Symbol	Gene Type	External Link	Genomic Location	#Samples with VIS (%Total Samples)	#HCC (%Total HCC)	#Non-Tumor (%Total Non-Tumor)	Odds Ratio	95% CI	*p*-Value
*TERT*	pc*	HGNC:11730	5p15.33	385 (8.44)	312 (12.75)	73 (3.45)	4.088	3.146–5.312	<0.01
*FN1*	pc*	HGNC:3778	2q35	278 (6.09)	29 (1.18)	249 (11.77)	0.09	0.061–0.133	<0.01
*KMT2B*	pc*	HGNC:15840	19q13.12	240 (5.26)	189 (7.72)	51 (2.41)	3.388	2.472–4.642	<0.01
*ALB*	pc*	HGNC:399	4q13.3	70 (1.53)	18 (0.74)	52 (2.46)	0.294	0.171–0.504	<0.01
*CCNA2*	pc*	HGNC:1578	4q27	36 (0.79)	28 (1.14)	8 (0.38)	3.049	1.387–6.704	<0.01
*LINC00486*	lncRNA	HGNC:42946	2p22.3	36 (0.79)	10 (0.41)	26 (1.23)	0.33	0.159–0.685	<0.01
*CPS1*	pc*	HGNC:2323	2q34	34 (0.74)	4 (0.16)	30 (1.42)	0.114	0.04–0.324	<0.01
*SH3RF3*	pc*	HGNC:24699	2q13	29 (0.64)	25 (1.02)	4 (0.19)	5.448	1.893–15.679	<0.01
*CCNE1*	pc*	HGNC:1589	19q12	27 (0.59)	22 (0.9)	5 (0.24)	3.829	1.447–10.128	<0.01
*KCNT2*	pc*	HGNC:18866	1q31.3	26 (0.57)	3 (0.12)	23 (1.09)	0.112	0.033–0.372	<0.01
*GLI2*	pc*	HGNC:4318	2q14.2	23 (0.5)	21 (0.86)	2 (0.09)	9.146	2.142–39.052	<0.01
*FAM157A*	lncRNA	HGNC:34079	3q29	23 (0.5)	22 (0.9)	1 (0.05)	19.18	2.583–142.416	<0.01
*GTF2I*	pc*	HGNC:4659	7q11.23	22 (0.48)	1 (0.04)	21 (0.99)	0.041	0.005–0.303	<0.01
*CHRM3*	pc*	HGNC:1952	1q43	21 (0.46)	1 (0.04)	20 (0.95)	0.043	0.006–0.319	<0.01
*PCMTD2*	pc*	HGNC:15882	20q13.33	20 (0.44)	18 (0.74)	2 (0.09)	7.83	1.815–33.784	<0.01

*pc = protein-coding.

## Data Availability

Data supporting the reported results were obtained from the research papers numbered under the citation indexes [10,13,24,27,28,29,30,31,32,33,34,35,36,37,38,39,40,41,42,43,44,45,46,47,48,49,50,51,52,53] and the VISDB database [54].

## Abbreviations

The following abbreviations are used in this manuscript:

HBV	Hepatitis B virus
AHB	Acute HBV infection
CHB	Chronic HBV infection
HCC	Hepatocellular carcinoma
HBV-HCC	HBV-associated hepatocellular carcinoma
HBeAg	Hepatitis B e antigen
HBcAg	Hepatitis B core antigen
HBsAg	Hepatitis B surface antigen
anti-HBs	Hepatitis B surface antibody
anti-HBc	Total hepatitis B core antibody
IgM anti-HBc	IgM antibody to hepatitis B core antigen
